# The Sound of Safety: DIVOT (Doppler Imaging for Vascular Orientation in Thoracic Procedures) Protocol

**DOI:** 10.24908/pocusj.v10i01.18071

**Published:** 2025-04-15

**Authors:** Amy Fraser, Daniel S. Brenner, Matthew Coghlan, Heather Andrade, Maya Haouili, William Graham Carlos, Edwin Jackson

**Affiliations:** 1 Internal Medicine Residency Program, Indiana University School of Medicine, Indianapolis, IN, USA; 2 Department of Emergency Medicine, Indiana University School of Medicine, Indianapolis, IN, USA; 3 Division of Pulmonary and Critical Care Medicine, Indiana University School of Medicine, Indianapolis, IN, USA; 4 Internal Medicine-Pediatrics Residency Program, Indiana University School of Medicine, Indianapolis, IN, USA

**Keywords:** thoracenteisis, doppler, ultrasound, thoracostomy, intercostal arteries

## Abstract

Each year, more than 200,000 thoracentesis and percutaneous chest tube thoracostomy procedures are performed in the United States [1–4]. In both procedures, the initial step involves advancing a needle over the superior aspect of the rib into the intercostal space to access the pleural cavity. Traditional teaching suggests that this technique avoids the neurovascular bundle, which is typically shielded by the inferior border of the rib. However, this technique does not guarantee safety. Computed tomography studies have shown that the intercostal arteries (ICAs) are highly tortuous, with positions that can vary significantly within the intercostal space [5–7]. This variability can lead to ICA laceration even with an optimal traditional technique [8–9]. Significant hemorrhage into the pleural space may initially go unnoticed but can progress to hemorrhagic shock or even tension hemothorax physiology [10–12]. Improved procedural guidance is needed to enhance safety and achieve the goal of zero patient harm. We propose the DIVOT (Doppler Imaging for Vascular Orientation in Thoracic procedures) protocol using a combination of high-frequency linear ultrasound, color, and Power Doppler (PD) to identify an ICA and its collaterals before needle insertion. This can reduce the risk of accidental vascular injury during thoracentesis or percutaneous chest tube thoracostomy.

## Background

**Figure 1.**
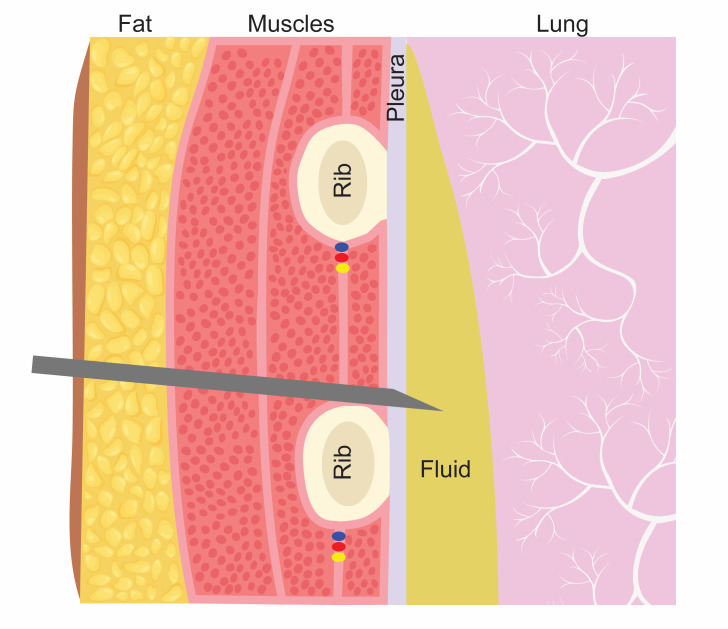
Traditional recommendation for thoracentesis: Needle over the rib to avoid the neurovascular bundle.

Thoracentesis and percutaneous chest tube insertion are common procedures that can be associated with serious adverse events. The most dangerous of these complications is laceration of the intercostal artery (ICA) with subsequent hemothorax [1, 6, 8–9], which has been shown to occur in up to 2% of non-intubated patients, 3.3% of mechanically ventilated patients [2, 3, 8–10], and up to 3% of patients on therapeutic anticoagulation [11]. Current guidelines for thoracentesis and percutaneous tube thoracostomy recommend avoiding the neurovascular bundle by inserting the needle over the superior aspect of the rib to access the pleural space (Figure 1) [13].

Despite using this technique, laceration of the ICA can still occur, leading to life-threatening pleural hemorrhage. This hemorrhage is especially difficult to control due to the combination of negative pressure in the pleural cavity and the difficulty of applying external compression to the bleeding vessel. As a result, ICA lacerations often require surgical repair, increasing both cost and morbidity [1, 3, 8–10, 14]. We believe that visualizing the ICAs and their collateral branches can reduce complications such as arterial laceration and intrapleural hemorrhage [14]. Two-dimensional ultrasound guidance significantly improves success rates and reduces the incidence of pneumothorax during pleural procedures [1] and may reduce the risk of hemothorax [10]. However, it cannot fully prevent damage to the ICA. This limitation arises because two-dimensional ultrasound cannot detect blood flow, making it difficult to visualize and avoid the ICA during needle insertion. To address this limitation, we present the DIVOT (Doppler Imaging for Vascular Orientation in Thoracic procedures) protocol, which combines two-dimensional imaging with color and Power Doppler (PD) techniques. Although hemorrhagic complications from thoracentesis and percutaneous chest tube thoracostomy are rare, even a single preventable incident is unacceptable in the current era of zero-harm initiatives. This protocol provides comprehensive procedural guidance to minimize the risk of hemorrhagic complications associated with thoracentesis and percutaneous chest tube placement, supporting our commitment to eliminating preventable harm.

## Ultrasound-Guided Thoracentesis Protocol

**Figure 2.**
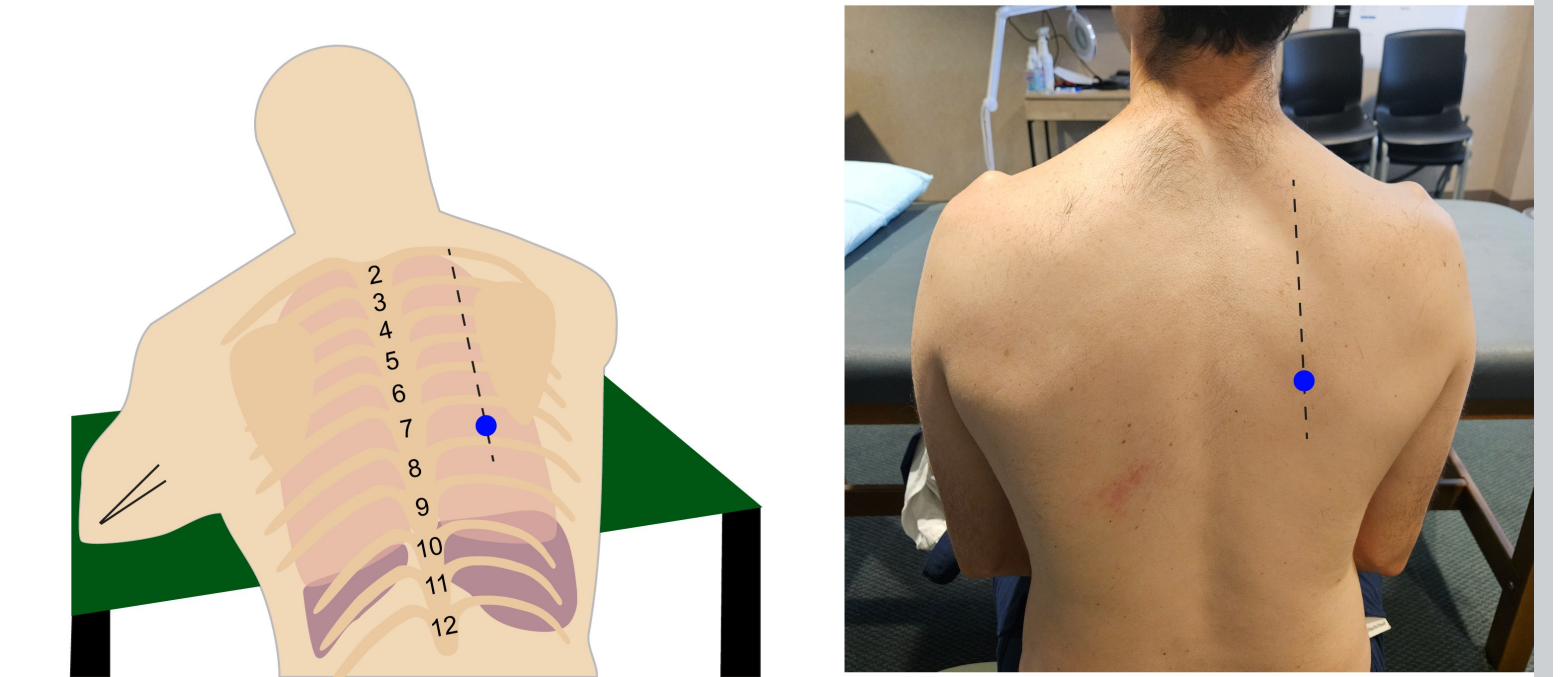
Seated positioning for pleural access.

**Figure 3.**
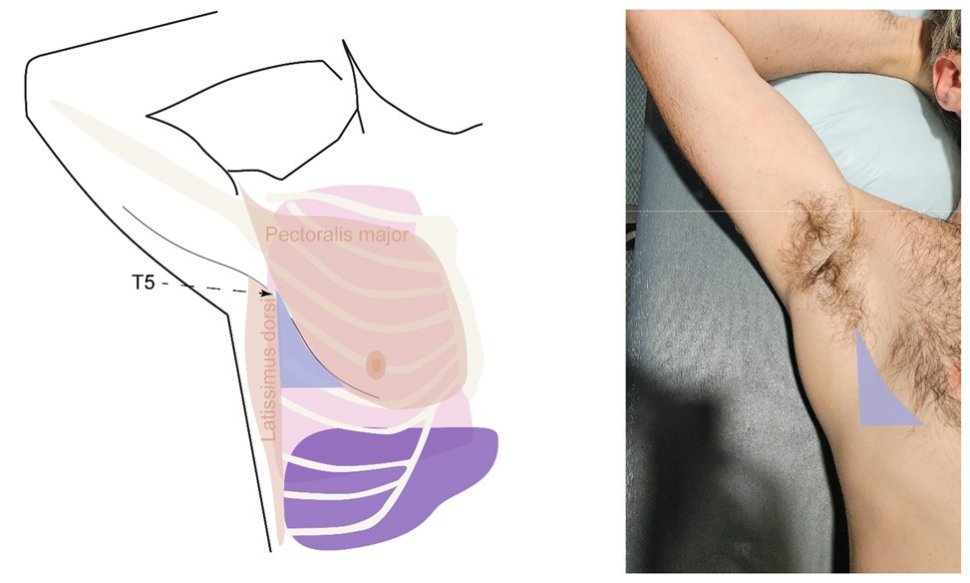
Supine triangle of safety position. The blue shaded area is the recommended insertion zone.

In clinical practice, the seated position is commonly used for pleural access in non-intubated patients. In this position, the patient sits upright or leans slightly forward with their arms resting on a table, allowing optimal access to the pleural space and facilitating the use of ultrasound guidance (Figure 2). For intubated or immobile patients, the supine position is typically preferred, targeting access through the “triangle of safety.” This anatomical region is bounded by the lateral edge of the pectoralis major muscle anteriorly, the lateral edge of the latissimus dorsi muscle posteriorly, and a horizontal line at the fifth intercostal space (aligned with the nipple) inferiorly (Figure 3).

It is important to note that in either of these positions, the ICA can have an unpredictable course and may be located above the rib within the area of needle insertion.

## DIVOT Protocol

Video S1 demonstrates the full protocol outlined below.

### Step 1: Initial Evaluation

Using a curvilinear or phased array transducer, locate the area of interest and document the rib space, depth, and character of pleural fluid. Next, mark this area with a skin marking pen for subsequent identification during Step 2.

### Step 2: Detailed Assessment

**Figure 4.**
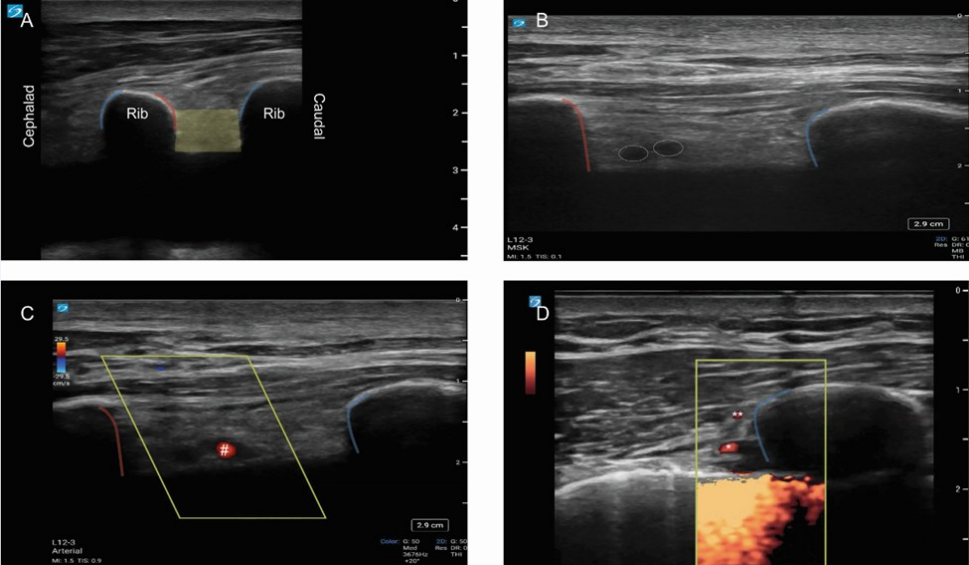
A) Identified superior (blue outline) and inferior (red outline) borders of the rib along with the intercostal space (shaded yellow). B) Rounded hypoechoic structures beneath the rib (intercostal artery (ICA) & intercostal vein). C) ICA identified by color Doppler (red). D) Power Doppler.

**A**: Position the leading edge of a high-frequency linear transducer at the designated needle insertion site identified in Step 1. Once the site is verified, align the transducer over the area, ensuring the intercostal space, along with the ribs bordering it superiorly and inferiorly, is distinctly visible. Orient the transducer indicator cranially (in the sagittal plane) and situate it perpendicular to the ribs and intercostal space. Adjust the depth setting to adequately delineate the intercostal structures, typically at least 4 cm. Identify the superior border of the rib (blue line), the inferior border (red line), and the intercostal space (yellow space) (Figure 4A). In some cases, hypoechoic structures can be identified below the inferior border of the rib, as depicted in Figure 4B, which is the typical location of the ICA and vein. However, it is common for vessels in this area to remain unseen because the ICA may be hidden within the intercostal groove, reducing ultrasound visibility.

**B**: Utilize the zoom function to magnify the intercostal space as well as the margins of the adjacent ribs. Next, activate Color Doppler Imaging (CDI) and position the color box from the inferior edge of the superior rib to the superior border of the inferior rib, extending across the entire intercostal space to the level of the pleural or pleural fluid. Set the flow scale to an initial range of 8–10 cm/s and decrease incrementally (e.g., by 2 cm/s) until optimal visualization of the ICA is achieved. The ICA should appear red and pulsatile. At this stage, confirm that your color Doppler settings, such as color gain, are optimized to avoid artifacts and ensure that any visible vasculature is identified.

**C**: As a final safety check, activate PD to detect collateral vessels with low-flow velocities that may have been neglected, particularly at the site of proposed needle insertion. The PD box should be positioned above and below the rib (Figure 4D). Refinement of PD settings can be performed by carefully adjusting the following:

1. PD Box Size: Size the PD box to encompass the intercostal space along with the superior and inferior portions of the adjacent ribs, ensuring adequate coverage without compromising sensitivity.
2. PD Gain: Optimize the signal by gradually increasing the gain until noise artifacts become visible, then reducing it slightly until the artifacts are no longer present.
3. Pulse Repetition Frequency (PRF): Adjust the PRF to detect low-flow vessels while avoiding excessive aliasing or flash artifacts. A typical range for intercostal vessels is 300–500 Hz.
4. Motion Artifacts: Ensure transducer stabilization by anchoring your hand and minimizing patient movement.

If these collateral vessels are close to the site of needle insertion, this finding should prompt the evaluation of a different rib space for needle insertion.

**D**: If no vessels are visualized using PD, the transducer should be rotated 90° within the same rib space, shifting from a sagittal to a transverse orientation. Gradually fan the transducer cephalad and caudad to fully assess the superior border of the rib at the proposed needle entry site. Pause midway through this motion and activate PD to confirm that no vessels cross the targeted area. This precaution is critical as the proceduralist prepares to apply the ‘rib-walking’ technique, deliberately contacting the rib to guide the needle safely over its superior border. If the area is clear of vessels, needle insertion at this location can be confidently performed.

## Conclusion

Thoracentesis and percutaneous tube thoracostomy are commonly performed procedures with established protocols to minimize complications using two-dimensional ultrasound. However, the risk of ICA injury remains a significant concern that two-dimensional ultrasound alone cannot fully address. The DIVOT protocol offers a comprehensive approach for assessing ICAs and collateral vessels before needle insertion. Previous studies have established the feasibility of ICA screening. Bedawi et al. demonstrated that using a low-frequency curvilinear transducer scanning a single position allowed for ICA screening. This approach identified the ICA in 52.9% of cases, resulting in a change of procedure site in 15.6% of cases and abandonment of the procedure in only 1.2% [17]. However, the study did not employ a high-frequency transducer or PD, raising concerns about the potential for missed detection of smaller or more tortuous intercostal collateral vessels. In a separate study by Salamonsen et al., patients underwent both high-frequency linear ultrasound and arterial-phase contrast-enhanced computed tomography. Ultrasound demonstrated an 88% sensitivity for detecting the ICA in the intercostal space when compared with the computed tomography gold standard [18].

At our institution, we have implemented variations of the DIVOT protocol in thoracentesis and percutaneous tube thoracostomy cases. The DIVOT protocol seamlessly integrates into the pre-procedural scanning already performed before pleural procedures. In experienced hands, the protocol adds only two minutes to the procedure and can be effectively taught to novices in a single session. Ongoing studies are assessing the feasibility of teaching the protocol to medical students, residents, nurse practitioners, and physicians to expand its application in clinical practice. In conclusion, the implementation of the DIVOT may enhance procedural safety and precision with minimal added time. The DIVOT protocol holds promise for widespread adoption, ultimately advancing our goal of achieving zero harm.


